# Empathy affects tradeoffs between life's quality and duration

**DOI:** 10.1371/journal.pone.0221652

**Published:** 2019-10-24

**Authors:** Adrianna C. Jenkins

**Affiliations:** Department of Psychology, University of Pennsylvania, Philadelphia, Pennsylvania, United States of America; University of Amsterdam, NETHERLANDS

## Abstract

Sharing others’ emotional experience through empathy has been widely linked to prosocial behavior, i.e., behavior that aims to improve others’ welfare. However, different aspects of a person’s welfare do not always move in concert. The present research investigated how empathy affects tradeoffs between two different aspects of others’ welfare: their experience (quality of life) and existence (duration of life). Three experiments offer evidence that empathy increases the priority people place on reducing others’ suffering relative to prolonging their lives. Participants assigned to high or low empathy conditions considered scenarios in which saving a person’s life was incompatible with extinguishing the person’s suffering. Higher empathy for a suffering accident victim was associated with greater preference to let the person die rather than keep the person alive. Participants expressed greater preference to end the lives of friends than strangers (Experiment 1), those whose perspectives they had taken than those whom they considered from afar (Experiment 2), and those who remained alert and actively suffering than those whose injuries had rendered them unconscious (Experiment 3). These results highlight a distinction between empathy’s effects on the motivation to reduce another person’s suffering and its effects on the prosocial behaviors that sometimes, but do not necessarily, follow from that motivation, including saving the person’s life. Results have implications for scientific understanding of the relationship between empathy and morality and for contexts in which people make decisions on behalf of others.

## Introduction

Across human societies and multiple species, individuals have been found to privilege the welfare of close others. Whether closeness derives from kinship [1], similarity [2], or shared group membership [3,4], individuals tend to engage in more helpful behaviors toward socially close than socially distant recipients [5–11].

In humans, it is hypothesized that the behavioral tendency to prioritize the welfare of close others is driven, at least in part, by a psychological tendency to empathize preferentially with close others [2,4,12,13]—i.e., to experience emotional states congruent with their situations [9,14–16]. The more one resonates with another’s emotional experience, it is thought, the more motivated one will be to improve that experience [9,15,17–20]. Although definitions of empathy differ [21], evidence suggests that sensorimotor [22,23], affective [24–28], and cognitive [29–31] aspects of one individual’s response to another can all be modulated by the degree of closeness between the two individuals, with more robust responses elicited by close others [32]. Similarly, empathic responses can be increased by manipulations that deliberately promote the closeness between the perceiver and target, such as perspective-taking [2,33–35].

In turn, empathizing with another person is widely associated with helpful behaviors such as donating to that person’s cause [36,37] or rescuing the person from a painful situation [17,38,39]. Individuals higher in trait empathy generally tend to help others more [40,41], whereas failures to empathize have been associated with curtailed helping and even a propensity to cause others harm [42–46]. Together, these observations support the idea that closeness promotes empathy, which generally promotes prosocial behavior, i.e., behavior that aims to improve others’ welfare [47].

However, different aspects of another person’s welfare do not always move in concert. A rigorous medical treatment might prolong a patient’s life but cause her to suffer, and dangerous or unhealthy habits might shorten a person’s life but fill him with glee. Cases like these capture the possibility for tension between behaviors that would promote two aspects of a person’s welfare: those that would promote a person’s subjective, psychological *experience* (quality of life) by reducing pain and suffering or increasing pleasure and happiness, and those that would promote a person’s objective, physical *existence* (duration of life), by increasing physical health or longevity.

To date, research on empathy has typically considered cases in which changes to others’ experience and existence move in tandem or are otherwise compatible. For example, rescuing drowning children [20] has the potential to both reduce their discomfort and keep them alive, and rescuing strangers from a mild electric shock [17] could protect them from pain without consequences for their life expectancy. It is on the basis of situations like these that closeness and empathy are generally understood to motivate promoting others’ welfare. Yet, it is in also these situations that a motivation to reduce others’ suffering could bring along other prosocial outcomes for the ride. When reducing suffering can be accomplished by conventional forms of helping, empathy appears to promote those conventional forms of helping. In contrast, little is known about the consequences of closeness, and the empathy associated with it, when actions that would promote different aspects of another person’s welfare are misaligned.

Here, we investigated how social closeness and the empathy that follows from it affect people’s preferences for different outcomes when outcomes that would promote others’ experience (extinguish suffering) and existence (prolong life) are mutually incompatible. In these cases, an anticipated change in another person’s psychological experience is not a good proxy for an anticipated change in other aspects of their welfare, making it possible to explore the possibility that the link between empathy for a suffering person and prosocial behavior hinges on the behavior’s compatibility with extinguishing the person’s suffering. When the only means to extinguish a person’s suffering was to end that person’s life, we predicted that higher empathy for a suffering person would be associated with motivations that resemble those typically associated with failures to empathize: greater preference to let the person die. In turn, to the extent that people empathize more strongly with close than distant others, we predicted that people would express greater desire to save the lives of suffering distant others than suffering close others. (A parallel set of predictions concerns the consequences of empathizing with another person’s positive experience, or pleasure [48], which we leave to future study.)

## General method

Participants in all studies provided written informed consent and participated in a manner approved by the Committee on the Use of Human Subjects at Harvard University (protocol #17791).

To examine tradeoffs between the quality and duration of other lives, we aimed to create ecologically plausible situations in which (i) experience and existence concerns were both at stake and (ii) the actions that would promote them were mutually incompatible. Accordingly, in three experiments, we exposed perceivers to scenarios describing a target person in an extremely painful, life-threatening situation (see S1 File for full details). In each experiment, we assigned each participant to either a higher or a lower empathy condition, manipulated between-subjects. In Experiment 1, we manipulated the interpersonal closeness between the participant and the protagonist (friend or stranger). In Experiment 2, we manipulated the perspective taken on the protagonist (close or distant). In Experiment 3, we manipulated the consciousness of the protagonist (conscious or unconscious). In all cases, participants read a scenario describing an accident and were asked to what extent it would be better for the person to be rescued (prolonging suffering but prolonging life) or to die in the accident (extinguishing suffering but ending life).

In order to isolate, as much as possible, the effect of empathy on participants’ preferences for what should happen to the victim, we did not ask which outcome participants would personally take action to bring about. Instead, we asked which outcome was better overall. Individuals’ own actions are subject to a wide variety of motivations over and above their preferences for what should happen to others, including motivations to act morally, follow through on professional responsibilities, abide by the law, and avoid harm to oneself. Accordingly, we asked, “all things considered, which is better…?” where both the life-saving and life-ending actions would be carried out by someone (or something) other than the participant.

Based on power analyses assuming two-tailed tests of a small-to-medium-sized effect with power > .80 and an alpha of *p* < .05 using G*Power, we aimed to collect data from 80–100 participants per condition per experiment [49]. In Experiment 1, recruitment was controlled by a computer program. In Experiments 2 and 3, data collection proceeded daily until at least 80 and no more than 100 individuals had participated in each condition (i.e., if fewer than 80 participants per condition had participated by the end of day n, data collection continued through the end of day n+1 unless the number of participants per condition reached 100 on that day, in which case data collection was stopped). All measures are reported.

## Experiment1: Social closeness

Experiment 1 investigated the effect of empathy on participants’ preference to save versus end a suffering person’s life by manipulating the social closeness between the participant and the victim.

### Method

#### Closeness manipulation

203 participants (95 female, 2 unreported; age range 18–83 years) were recruited online and assigned randomly to identify either a person they knew well and liked (*friend* condition; 101 participants) or one they had seen but never met (*stranger* condition; 102 participants) by entering the person’s initials into a free-response box on the computer screen. Participants then reported basic information about the target person, including the person’s age, gender, and the nature of their relationship.

#### Decision scenario

Next, participants read a vignette that described the nominated individual, identified by his or her initials, in a life-threatening and extremely painful accident. Participants in each condition were randomly assigned to read one of two possible scenarios (scenarios were designed to be interchangeable, and post-hoc analyses confirmed that neither participants’ empathy nor their preferences differed as a function of scenario type; both *p*’s > .25). In one scenario, the victim was described as suffering from a terminal illness and becoming trapped in an apartment building as it catches fire; in the other scenario, the victim was described as having a chronic but not terminal medical condition and experiencing a heart attack (see S1 File for details).

Participants in the friend and stranger conditions read vignettes that were identical in every way except that they included the initials and gender of the target person specified by the participant, thereby manipulating interpersonal closeness. For example, in the burning building scenario, a participant who nominated a female target with initials CC would have been asked to “Imagine that about a year ago, you learned that CC was diagnosed with a serious disease. After several tense discussions with doctors, CC told you that the disease was terminal; she didn’t know exactly how long she had to live […]. Since that time, CC’s condition has deteriorated rapidly, and she is in now constant, excruciating pain” (see Materials and Measures for full details). Next, the participant would have been asked to “imagine that it is the middle of the night, and an electrical fire has broken out in CC’s apartment. CC’s sheets have caught fire, and she is unable to move to escape. CC writhes helplessly in pain as the flames spread over her, severely burning her skin. The smoke begins to engulf her, and around her, she sees parts of the building begin to collapse.” For participants in the friend condition “CC” was someone close to them; for participants in the stranger condition, “CC” was someone they had seen before but had never met.

#### Life-or-death preference

Participants in both conditions reported the extent to which it would be better for the person to be rescued (coded as 7) or to die at that moment (coded as 1) using a continuous slider bar. For example, in the burning building scenario, participants were asked, “All things considered, would it be better if (A) CC is carried out of the building alive by the responding fire fighters or (B) CC is killed instantly by falling debris before the fire department arrives?”.

#### Empathy assessment

Participants responded to 6 questions assessing empathy: 3 other-focused questions (“How much pain would you say CC is feeling *right now*, as the building burns?”; “How much pain would you say CC was feeling *before the building caught fire*?”; “If she lives, how much pain is CC likely to feel *in the future*?”) and 3 self-focused questions (How much did you, yourself, feel CC’s pain while thinking about these events?”; “How sorry did you feel for CC while thinking about these events?”; “How uncomfortable was it for you to think about these events?”) on continuous scales from “very little” to “a tremendous amount” (converted to values between 1 and 7 for analysis) in random order. We observed a high degree of convergence among the six measures (Cronbach’s α = .70); accordingly, we averaged responses into a composite empathic emotion score with a maximum of 7 for each participant. (Exploratory follow-up analyses reported below also investigate possible roles for self-oriented and other-oriented emotion separately.) Participants were then asked to describe the reasons for their preference (free response) and provide basic demographic information on a final questionnaire.

### Results

#### Closeness decreased preferences to save a suffering person’s life

**Fig 1:**
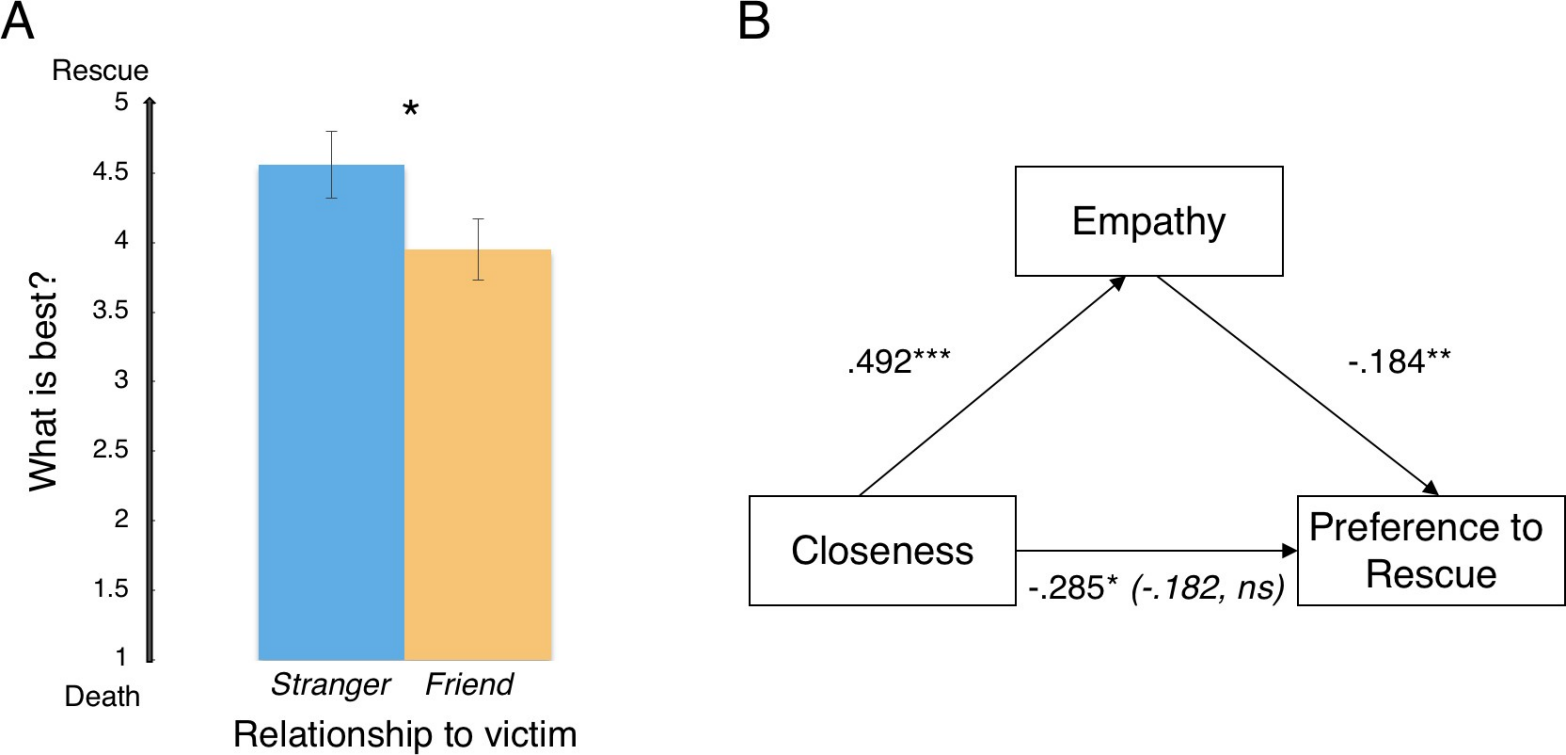
Results of Experiment 1. *Panel A*. Participants who considered a stranger in a painful, life-threatening situation expressed greater preference to save the person’s life than those who considered a friend in the same situation. *Panel B*. The relationship between closeness and the preference (not) to rescue was mediated by empathy, with higher empathy leading to lower rescuing preference. Standardized regression coefficients are indicated. Parentheses designate the standardized regression coefficient for the relationship between closeness and decision when controlling for empathic emotion; the relationship is no longer significant (*p*>.2). Condition was dummy-coded: stranger = 0, friend = 1. Error bars represent SEM. *p < .05; **p < .01. *** *p* < .001.

Participants expressed a significantly greater preference to rescue suffering strangers (*M* = 4.56) than suffering friends (*M* = 3.95) from a life-threatening accident, *t*(201) = 2.04, *p* < .042, *d* = 0.29, 95% CI [0.02, 1.19] (**Fig 1A)**.

#### Empathy mediated the relationship between closeness and the preference to let the person die

Consistent with the intense nature of the scenarios, participants on average reported a fairly high degree of empathic emotion (*M* = 4.91 on a 7-point scale). We also observed a significant effect of closeness on empathy: participants in the *friend* condition reported significantly more empathic emotion (*M* = 5.10) than participants in the *stranger* condition (*M* = 4.74), *t*(199) = 3.59, *p* = .0004, *d =* 0.51, 95% CI [1.13, 3.90].

The relationship between closeness and life-or-death preference was mediated by empathic emotion (**Fig 1B**). Participants who thought about a suffering friend (coded as 1) reported more empathic emotion than those who thought about a suffering stranger (coded as 0), *β* = .492, *p* < .001, and greater empathic emotion was associated with lower desire for the victim to be rescued, *β* = -.184, *p* < .01. That is, greater empathic emotion was associated with greater desire to end the victim’s life. When controlling for empathic emotion, the relationship between closeness and decision is no longer significant.

#### Examining a role for personal distress

There are at least two possible routes through which emotional responses to another person’s situation might increase the preference to end that person’s life. Previous work has distinguished personal distress, a self-oriented emotional response associated with escaping the aversive situation, from empathic concern (or compassion), an other-oriented emotional response associated with improving the target’s welfare [39, 50, 51, 52]. Following this distinction, we investigated the possibility that participants experiencing higher levels of empathic emotion overall might have been motivated primarily to reduce their own distress, such that their preference for the victim to die could reflect a motivation to escape psychologically from the situation. On a distress-based account, the intensity of participants’ own feelings of discomfort (more than the intensity of their attributions of discomfort to the victim) should be associated with the preference to let the victim die.

Analysis did not support a personal distress-based account of the current findings. Submitting the six emotion measures to principal components factor analysis (varimax rotation) did reveal two factors with eigenvalues over 1.0, which we termed Self-oriented emotion (how sorry participants felt, how much they ‘felt’ the pain of the target, and how upsetting they found the situation; eigenvalue = 1.32; 22.1% of variance) and Other-oriented emotion (victim’s initial pain, victim’s current pain, and victim’s future pain; eigenvalue = 2.42; 40.3% of variance). However, multiple mediation analysis [53] showed that Self-oriented emotion was associated with greater preference for rescue (b path: coeff = .11, *t* = 2.66, *p* < .01). In contrast, Other-oriented emotion was associated with greater preference to let the victim die (b path: coeff = -.31, *t* = 6.02, *p* < .0001). To the extent that self- and other- oriented emotion can be dissociated, these findings suggest that it was other-oriented empathy, rather than self-oriented distress, that primarily motivated letting a suffering person die in this context.

#### Free-responses underscore the motivation to end close others’ suffering

To gain further insight into the motivations for participants’ decisions using exploratory analyses, two independent readers, blind to condition, coded each free-response for the degree to which it reflected consideration of each of 13 possible factors: (1) the victim’s current suffering, (2) the victim’s future suffering, (3) the participant’s own feelings, (4) the feelings of someone other than the participant or victim, (5) the victim’s rights, (6) moral responsibility, (7) the inherent value of life, (8) God or religious beliefs, (9) the idea that the victim should choose for him- or herself, (10) the need for more information, (11) the victim’s personal history (e.g., having done something good/bad in the past), (12) what the participant would want if s/he were the victim, and (13) the participant’s personal desire not to lose the victim (see Table 1 for free-response examples). For each response, each dimension was coded 1 if it was not mentioned, 2 if it was mentioned but not primary, and 3 if it was the primary stated reason for the decision. Coders demonstrated a high degree of inter-rater reliability (mean two-way intra-class correlation [ICC] for ordinal data using absolute agreement across dimensions = .77, which falls within the highest, “excellent” range [54,55]. Accordingly, we averaged coders’ ratings for each response.

**Table 1:** Examples of participants’ explanations of their decisions in Experiment 1.

Condition	Participant Decision Explanation
**CLOSE** A close friend	Wishing her a quick, painless death is the only humane response a person can have.
	[Letting XX die] would minimize the amount of pain that he feels.
	I would want him to be free of pain, suffering and misery.
	Trying to imagine a world without her is difficult, so choosing to have her saved is better for me then to have her dead, but at the same time I don't want her to suffer any longer.
**DISTANT** Someone you have seen but never met	Pain shouldn't take away the beauty of life.
	[XX] has three little children and it would be very difficult for them to lose their mother.
	You can put an animal down but not a person.
	I'd like to know whether she gets any pleasure from spending time with family and what her wishes are to make a more representative decision.

Four of the thirteen factors were significantly associated with participants’ decisions when Bonferroni correcting for multiple comparisons. Specifically, the more strongly participants favored letting the victim die, the more likely they were to mention the victim’s current suffering, *r*(203) = .48, *p* < .001, the victim’s anticipated future suffering, *r*(203) = .50, *p* < .001, and the participant’s own empathic feelings, *r*(203) = .30, *p* < .001 when explaining the decision. Conversely, the more participants favored saving the victim’s life, the more likely they were to mention religious beliefs *r*(203) = .30, *p* < .001. Although correlational, these results offer tentative further support for the possibility that the desire to end a victim’s life was motivated by a desire to end the victim’s suffering.

## Experiment 2: Perspective-taking

The results of Experiment 1 suggest that interpersonal closeness shifted the priority participants placed on extinguishing another person’s suffering, relative to prolonging that person’s life, and identified a possible role for empathy in this shift. However, a number of differences between the two conditions in Experiment 1 limit the strength of the conclusions can be drawn about a specific role of empathy in these decisions, even though empathy was found to mediate the relationship. In particular, because participants in the two conditions considered different target individuals who differed in their relationship to the participant, factors other than the strength of the empathic response presumably distinguished the decision-making process across conditions and could have contributed to the observed differences in preference. To better isolate a role for empathy in tradeoffs between life’s quality and duration, Experiment 2 manipulated perceivers’ empathy for a single target individual using a well-established intervention known to affect empathy: perspective-taking [2,33–35,56,57].

In Experiment 2, participants either imagined what the victim would see, feel, and hear as the events took place (close condition) or imagined watching the events from above (distant condition) and then expressed a preference for what should happen to that person as in Experiment 1 (see S1 File for details). When actions that would promote experience and existence are compatible, imagining the experience of another person has been found to increase empathy and, in turn, prosocial behavior [51,58]. In cases of conflict between actions that would promote experience and existence, we predicted that participants who took the victim’s perspective would feel more empathic emotion, but express less inclination to save the victim’s life, than participants who considered the events from afar.

### Method

220 participants (124 female; age range 18–86 years) recruited around a nearby subway station were randomly assigned either to imagine what a person would see, hear, and feel from a field view (*close perspective* condition; 108 participants) or to imagine watching events unfold from an aerial, observer view (*distant perspective* condition; 112 participants) and read a vignette in which that person became severely-injured in an accident as in Experiment 1. Participants then reported their preference that the target live or die as in Experiment 1.

Guided by the results of Experiment 1, participants also answered 1 question about the current emotional experience of the target person (“How much is [the target] suffering right now?”), 1 question about the target’s future suffering (“If [he/she] lives, how much is [the target] likely to suffer in the future?”], and 1 question about their own emotional experience (“How much do you, yourself, ‘feel’ [the target’s] pain?) on 1–7 scales, which were averaged into a composite empathic emotion measure with a maximum possible score of 7 for each participant.

### Results

#### Perspective-taking increased empathy

As in Experiment 1, participants reported a high degree of empathic emotion on average (*M* = 4.96). Moreover, participants in the *close perspective* condition reported significantly more empathic emotion (*M* = 5.15) than participants in the *distant perspective* condition (*M* = 4.79), *t*(216) = 2.21, *p* = .028, *d* = 0.30, 95% CI [0.04, 0.68].

#### Perspective-taking was associated with lower desire to save a suffering person’s life

**Fig 2:**
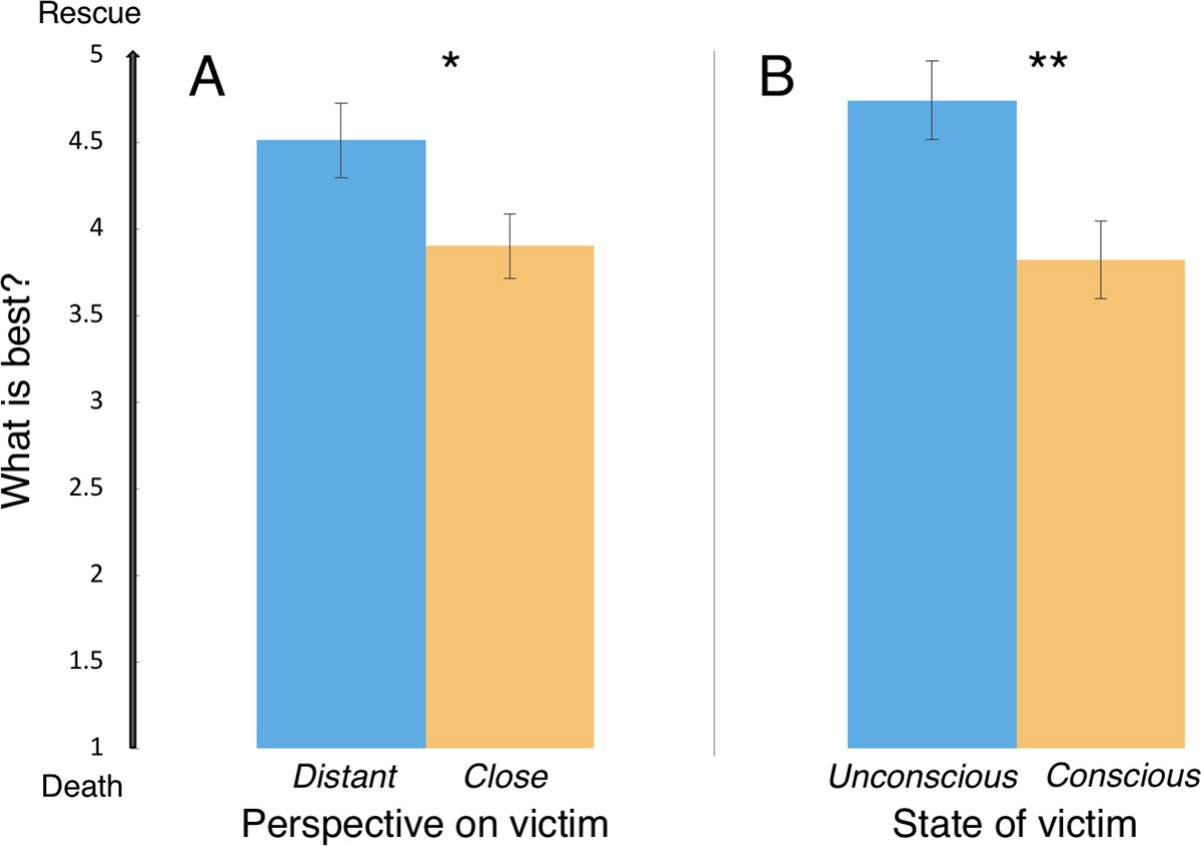
Results of Experiments 2 and 3. *A*. Participants who took a suffering victim’s perspective expressed less desire to save that person’s life than those who considered the victim from a distance. *B*. Participants expressed lower preference to save the life of a victim who remained actively suffering at the time of decision than one who temporarily lost consciousness. Error bars represent SEM. *p < .05; **p < .01.

Despite reading about the same targets in the same situations, participants in the *close perspective* condition expressed less desire to rescue the target (*M* = 3.90) than those in the *distant perspective* condition (*M* = 4.51), *t*(218) = 2.14, *p* = .033, *d* = 0.29, 95% CI [0.05, 1.17]; Fig 2A. That is, consistent with the possibility that empathy increases the motivation to extinguish suffering, relative to the desire to prolong life, participants who took the victim’s perspective reported more empathic emotion and were more likely to favor ending the victim’s life than participants who imagined the events from afar.

## Experiment 3: Consciousness

To the extent that the inclination to end a suffering accident victim’s life in Experiments 1 and 2 reflects a desire to extinguish the person’s suffering, perceivers should be less inclined to end the person’s life when suffering is extinguished by other means. To test this, Experiment 3 manipulated the consciousness of the injured individual immediately prior to participants’ decision in a between-subjects design. Holding constant the other circumstances of the person’s situation, Experiment 3 tested the prediction that perceivers would be less inclined to end a victim’s life when the victim’s suffering had been attenuated through a loss of consciousness than when the victim was conscious and actively suffering.

### Method

167 participants (97 female; age range 18–87) recruited around a nearby subway station read a vignette describing a life-threatening and extremely painful accident (see S1 File for details). Participants were randomly assigned to a condition in which the target remained alert in the height of suffering (*conscious victim* condition: 84 participants) or in which the target’s suffering was paused through a loss of consciousness (*unconscious victim* condition: 83 participants) at the end of the vignette.

Vignettes were identical up to the point of the consciousness manipulation.

For example, in the shark attack scenario, participants in the *conscious victim* condition read, “the shark thrashes sharply, and Dave feels the skin on one side of his face become loose as he is knocked sideways”, whereas participants in the *unconscious victim* condition read, “the shark thrashes sharply, and Dave feels the skin on one side of his face become loose as he is knocked unconscious.” Participants in both conditions were asked whether it would be better for the target person to die instantly or be rescued on a 1–7 scale as in Experiments 1 and 2.

### Results

#### Unconsciousness increased the desire to save an accident victim’s life

Participants who considered a victim who lost consciousness just before their decision expressed greater preference to have the victim rescued (*M* = 4.74) than participants who considered a victim who remained conscious in an otherwise identical situation (*M* = 3.82), *t*(162) = 2.83, *p* = .005, *d* = 0.44, 95% CI [0.28, 1.56]; Fig 2B. That is, participants expressed greater preference for an injured victim to die when that person remained conscious and actively suffering than when the person’s suffering had been paused through a loss of consciousness. This result is consistent with the possibility that participants’ greater preference to end the life of close friends (Experiment 1) and those whose perspective they had taken (Experiment 2) was driven by a greater desire extinguish those individuals’ suffering. When identical events transpired but the victim’s suffering was extinguished through a loss of consciousness just before their decision, participants expressed greater preference that the victim remain alive.

## Discussion

People are often encouraged to cultivate empathy toward others because doing so is expected to be better for those individuals. The present research explored the question, “better” in what sense? Participants faced hypothetical tradeoffs between two aspects of another person’s welfare: the quality and the duration of the person’s life. In otherwise identical situations, social distance and lower empathy were associated with greater preference for a suffering person to be rescued from an accident and continue to live. Social closeness and higher empathy were associated with greater preference for the victim to die on the spot, reflecting a greater desire to extinguish the victim’s suffering. These results caution against an oversimplified view of empathy in which it leads to better outcomes for a target person in a general sense. Instead, these results support the view that empathy motivates behaviors that are better in the specific sense that they aim to improve the target’s psychological experience—something that often, but not necessarily, goes hand in-hand with other desired outcomes.

More broadly, the current findings highlight the importance of distinguishing proximate from ultimate levels of explanation [59] when considering the motivational consequences of empathy. From an ultimate or evolutionary perspective, elevated empathic responses to the suffering of close others could serve adaptive functions by prompting actions that, on average, keep close others alive [1,14,60]. Because psychological suffering can often serve as a useful proxy for damage to other aspects of a person’s welfare, a person’s motivation to reduce close others’ suffering might, on average, have the effect of prolonging the lives of individuals who could propagate that person’s genes (e.g., children, siblings) [1] and/or reciprocate by helping the person survive some future mishap (e.g., friends, neighbors) [60], thereby increasing fitness. Yet, if, at a proximate level, empathy operates on psychological experience, then empathy will not necessarily lead to typical helping behaviors on any given occasion. In particular, elevated empathic responses to close others could appear to be maladaptive in instances when behaviors that would promote the other person’s existence and experience are misaligned.

These findings demonstrate that empathizing with another person can have detrimental effects on aspects of that same person’s welfare, joining other findings that empathizing with one person can have detrimental effects on the welfare of other people [61,62]. Specifically, past research has shown that empathizing with one person can lead the empathizer to privilege that person’s welfare over the welfare of other individuals with whom one has empathized less [63]. Whereas those studies suggest that empathy can motivate decisions that violate group-based moral principles (e.g., fairness), the current studies suggest that empathy can also motivate decisions that violate individual-based moral principles, at least as they are typically understood, including avoiding harm [64,65]. At the extreme, the current results suggest that empathy sometimes increases preferences for ending a person’s life. On some views, this would mean that empathy can increase people’s preference that the ultimate form of harm (death) befall the target of empathy him- or herself.

The current findings accordingly inform the relationship between empathy and morality. In past research, a role for harm aversion in moral decisions has been discussed at length [66–68], but whether empathy selectively increases an aversion to certain types of harm has not characterized. The current results help to clarify the scope of empathy-related harm aversion by suggesting that empathy may increase people’s aversion, first and foremost, to others’ affective or psychological harm—i.e., their pain and suffering. To the extent that empathy sometimes does motivate life-saving, it could be the case that empathy primarily increases the aversion, not to death itself, but to the suffering that often precedes it.

If empathizing with another person can have detrimental effects on that person’s welfare, why have these effects been mostly absent in past research? One possible explanation is that past research has predominantly studied decisions in which one course of action is uniformly better for the target person and another course of action is uniformly worse [69]. In these situations, benefits to the quality and duration of a person’s life move in tandem, and a motivation to improve another’s experience could bring with it benefits to the person’s existence. In contrast, the current studies focus on decisions in which the quality and duration of a person’s life are in conflict, suggesting that the link between empathy and the motivation to engage in prototypical helping behaviors (like saving another’s life), may hinge on the extent to which those behaviors are compatible with improving the person’s experience.

The use of hypothetical scenarios in the present work brings with it several limitations on generalizability. First, these scenarios were not meant to reflect decisions that typical individuals are likely to face in everyday life but rather to isolate certain factors in a way that made it possible to illuminate the consequences of empathic motivation. Just as individuals in daily life rarely name the color of ink in which a word is written, as they do in the Stroop task, or flip train switches, as they do in trolley dilemmas, participants are unlikely to find themselves in a position of deciding whether someone in a burning building should be rescued or die on the spot. By their extreme nature, these scenarios enabled us to create conditions in which extinguishing a person’s suffering and prolonging his or her life were incompatible, making it possible to capture shifts in people’s preferences toward one outcome at the cost of the other. Second, the current studies deliberately did not ask participants what they would do if they were personally involved in the situation. Additional research is needed to understand how the observed effect of empathy on participants’ preferences to end a suffering person’s life interacts with other motivations that guide behavior, including the motivation to be a moral person, to follow through on responsibilities, to avoid guilt, to abide by the law, and others.

Future research is needed to explore implications of the current findings for domains in which people regularly do make decisions on behalf of others, including medical decision-making, where a need for empathy is often cited [70–74]. In particular, if empathy increases the priority placed on a person’s experience, empathy may be more beneficial in some kinds of medical situations than in others. For example, empathy might help a physician perform an injection carefully or convey bad news sensitively; in cases like these, there is little conflict between behaviors that promote the quality and duration of a patient’s life. However, empathizing with a patient in a tradeoff situation between his or her experience and long-term health may tug physicians toward maximizing the quality of the patient’s experience in the moment rather than the number of moments in the patient’s future. This possibility is broadly consistent with past research on end-of-life decisions demonstrating that family members and nurses are more inclined to end the life of a terminally ill patient when the person has intractable pain [75] or is unable to carry out valued life activities [76]. In turn, these results suggest the tentative prediction, open to future research, that physicians with lower trait empathy might be more inclined to take courses of action to prolong a suffering patient’s life.

In interpreting these findings, it is worth noting that our experiments measured empathic emotion, or “affective empathy” [21]. As such, we anticipate that these findings will apply to other situations in which individuals empathize with a suffering person, as characterized by experiencing an affective state congruent with that person’s situation [9,15,16]. A subset of those experiences may also be accompanied by feelings of empathic concern—feelings of caring, compassion, or pity for the other person [47]—which could combine with the phenomenon observed here to guide a perceiver’s ultimate decision about which course of action to pursue [50]. Relatedly, future research will be needed to characterize more precisely the respective contributions to decision-making of (i) the strength of the empathic response to another person’s suffering itself and (ii) the strength of the motivation to help a suffering person with whom one has empathized.

These findings do not necessarily challenge the overall observation that empathy motivates improving others’ welfare. However, they do help to specify the kind of welfare that empathy motivates improving and point to some of the potential consequences of that motivation. In the realm of individual decision-making, it is well known that people’s affective responses can lead them to pursue immediately desirable psychological states (e.g., the pleasure of chocolate chip cookies) at the expense of other goals (e.g., maintaining a svelte figure). Similarly, empathizing with the affective experiences of other people might boost one’s motivation to prioritize the quality of those people’s internal states at the expense of other aspects of their welfare—including, at the extreme, the durations of their lives.

## Supporting information

S1 File Materials and measures. (PDF)
