# Supplementary material for: Empathy affects tradeoffs between life's quality and duration
Source: PLoS One. 2019 Oct 24;14(10):e0221652. doi: 10.1371/journal.pone.0221652 (PMC6812864; doi:10.1371/journal.pone.0221652)
Supplement: S1 File — (PDF) [file pone.0221652.s001.pdf]

S1 File
Materials and Measures
Empathy affects tradeoffs between life's quality and duration
A.C. Jenkins

Experiment 1: Closeness

1. Closeness manipulation. Participants were randomly assigned to either the close or distant condition and entered basic information about someone they knew well and liked (close) or someone they had seen before but didn't know personally (distant).

Close condition:

"Please take a moment to think of someone you know and like. This could be a friend, a co-worker, a teacher, or anyone else you know fairly well and like quite a bit -- but please think of one specific person."

"Enter the initials (first and last) of the person you have selected here: __"

"What is this person's relationship to you?"
Acquaintance
Friend
Sibling
Parent
Child
Spouse/Significant Other
Other (specify)

"How close are you to this person?"
1 2 3 4 5 6 7 (1=not at all close, 4= moderately close, 7=extremely close)

"Approximately how long have you known this person?"
1-6 months
6 months to 1 year
1-5 years
5-10 years
More than 10 years

"What is this person's gender?"
Male
Female

"What is this person's approximate age?"
0-15
16-30
31-45
46-60
61-75
76-90
91+

Distant condition:

"Please take a moment to think of someone whom you've seen before but whom you don't know personally. This could be someone you've seen a few times on a bus, someone you have a class with, or anyone else you don't personally know -- but please think of one specific person."

"Enter the initials (first and last) of the person you have selected here: __"
"Approximately how long ago was the first time you encountered this person?"
1-6 months
6 months to 1 year
1-5 years
5-10 years
More than 10 years

"What is this person's gender?"
Male
Female

"What is this person's approximate age?"
0-15
16-30
31-45
46-60
61-75
76-90
91+

"Under what circumstances have you encountered this person?" (free response)

2. ***Suffering situation.*** In both conditions, participants were randomly assigned to read one vignette about a person experiencing extreme suffering (either the "Burning building" or the "Hospital emergency" scenario; names of scenarios were not shown to participants). Scenarios were meant to be interchangeable; we did not have a priori hypotheses about the effect of vignette type and did not plan to analyze it as a factor. Post-hoc analyses showed that neither participants' empathy nor their decisions differed as a function of vignette type.

For each vignette, participants in the close and distant conditions saw exactly the same information, except that the initials and gender of the protagonist were those they had entered above (here indicated by XX).

Burning building scenario (seen by approximately half of the participants in each condition)

"On the following screens, you will be asked to imagine the person you have selected in a hypothetical scenario. Then you will be asked a few questions about the story."

“Imagine that about a year ago, you learned that XX was diagnosed with a serious disease. After several tense discussions with doctors, XX told you that the disease was terminal; [he/she] didn’t know exactly how long [he/she] had to live, but it wouldn’t be very long. Since that time, XX’s condition has deteriorated rapidly, and [he/she] is in now constant, excruciating pain. Frustratingly, the pain is only worsened when [he/she] tries to move, and, to make matters worse, [he/she] has lost most of [his/her] control of [his/her] muscles. XX hardly even resembles the person you knew before. [His/Her] daily existence is one of agony and helplessness, as the side effects of [his/her] medication, coupled with [his/her] symptoms, leave [him/her] bedridden and suffering. XX is visited by a nurse, who feeds [him/her] and checks on [his/her] symptoms each day, but [he/she] isn’t able to handle many other visitors. The doctors say XX’s condition will only continue to worsen until the disease takes over [his/her] body completely.”

“Imagine that it is the middle of the night, and an electrical fire has broken out in XX’s apartment complex, quickly engulfing the entire building in flames. XX’s sheets have caught fire, and [he/she] is unable to move to escape. [He/She] writhes helplessly in pain as the flames spread over [him/her], severely burning [his/her] skin. The smoke engulfs [him/her], and around [him/her], parts of the building begin to collapse.”

Hospital Emergency Scenario (seen by approximately half of the participants in each condition)

“On the following screens, you will be asked to imagine the person you have selected in a hypothetical scenario. Then you will be asked a few questions about the story.”

“Imagine that about two years ago, everything changed for XX when [she/he] was diagnosed with life-threatening blood disease. By the time it was diagnosed, the disease had already damaged much of [his/her] body. XX immediately began a course of rigorous hospital treatments, but [his/her] condition deteriorated rapidly. [She/He] experienced several organ failures, each followed by long, painful, and exhausting periods of attempted recovery. Today, XX’s body is so damaged that [he/she] can no longer walk. [She/He] is confined to a hospital bed, attached to several machines. When [he/she] is awake, XX experiences an excruciating, burning sensation, as though someone were peeling off [his/her] skin. XX’s condition is terminal; there is no known treatment, and doctors estimate that [he/she] doesn’t have long to live.”

“Imagine that early this morning, XX has a severe heart attack that requires immediate surgery. XX is rushed to the operating room, but the heart-and-lung machine has malfunctioned and the operating team cannot get the machine to work. All of the other machines in the hospital are in use. Writhing in pain, Andy looks around at the doctors. If the technician does not arrive in a couple of minutes to fix the machine, XX will die.”

3. ***Life-or-death decision.*** Participants responded using a continuous slider bar anchored by “A is much better” and “B is much better”.

Burning building scenario
“All things considered, would it be better if
(A) XX is carried out of the building alive by the responding fire fighters
or

(B) XX is killed instantly by falling debris before the fire department arrives?”

Hospital emergency scenario
“All things considered, would it be better if
(A) A technician arrives in time to repair the equipment and XX continues living or
(B) A technician does not arrive in time to repair the machine and XX dies?”

4. Emotion assessment. Questions were asked in random order.

"How much pain would you say [initials] is feeling *right now, as the building burns [right now, on the operating table]*?"
Very little;A moderate amount;A tremendous amount

“How much pain would you say [initials] was feeling *before the building caught fire [before the heart attack]*?”
Very little;A moderate amount;A tremendous amount

"If [he/she] lives, how much pain is [initials] likely to feel *in the future*?"
Very little;A moderate amount;A tremendous amount

"How much did you, yourself, feel [initials]'s pain while thinking about these events?"
Very little;A moderate amount;A tremendous amount

“How sorry did you feel for [initials] while thinking about these events?”
Very little;A moderate amount;A tremendous amount

“How uncomfortable was it for you to think about these events?”
Very little;A moderate amount;A tremendous amount

5. Decision Explanation.

“If you have any comments or would like to share why you made the choice you did about whether or not XX should continue to live, please use this box:”

6. Demographic questionnaire.

“Thank you for your participation so far. We just have a few more quick questions for you. Please answer honestly and accurately.”

How much formal education have you completed?
Less than high school
High school diploma
Some college
BA/BS degree
BA plus some graduate work
Graduate degree

How would you describe your ethnicity?
American Native/Alaskan Native
Asian/Pacific Islander

Hispanic
White non-Hispanic
Black non-Hispanic
Multiracial
Other
Other "If you selected ‘Other ethnicity’, please name:

"With what religion, if any, were you raised?"
Buddhist
Christian (Orthodox)
Christian (Catholic)
Christian (Protestant)
Christian (Other)
Hindu
Jewish
Muslim
Sikh
None
Other

"If you selected “Other”, please name:"

"With what religious organization, if any, do you identify now?"
Buddhist
Christian (Orthodox)
Christian (Catholic)
Christian (Protestant)
Christian (Other)
Hindu
Jewish
Muslim
Sikh
None
Other

"If you selected “Other”, please name:"

In what country do you currently live?
[pulldown list]

Is English your native language?
No
Yes

If answered no, “what is your native language?"

Are you currently married?
No

Yes

How many children do you have?

What is your occupation?
Administrative support
Education: post-secondary
Education: primary, secondary
Education: other
Faith-related work
First responder: police, fire department, other
Health care
Home and family
Legal work
Manufacturing
Military
Non-profit
Public policy
Student
Services or personal care
Unemployed
Other"
“If you selected “Other”, please name:"

What is your approximate annual income?
"$0-$10,000
10,000-$20,000
$20,000-$30,000
$30,000-$40,000
$40,000-$50,000
$50,000-$60,000
$60,000-$70,000
$70,000-$80,000
$80,000-$90,000
$90,000-$100,000
$100,000-$110,000
$110,000-$120,000
$120,000-$130,000
$130,000-$140,000
$140,000-$150,000
$150,000+"

"When it comes to politics, how liberal or conservative are you?"
1-7 scale anchored by “Very liberal”, “Very conservative”

Have you ever had a pet?
No
Dog
Cat
Rabbit
Bird

Hamster
Horse
Snake
Fish
Other
"If you selected “Other”, please name:"

Have you ever known someone well who was terminally ill?
No
Yes

Has someone you knew well died? (e.g. a family member or close friend)
No
Yes

"If someone you knew well has died, about how long ago was the most recent time this happened? Please indicate number of days, months, or years."

"Do you believe in an afterlife? (e.g. heaven, hell, purgatory, reincarnation, etc.)"
1-7 scale anchored by “Definitely”, “Not at all”

"All things considered, how happy are you with your life?"
1-7 scale anchored by “Extremely unhappy”, “Neither happy nor unhappy”, “Extremely happy”

Experiment 2: Perspective-taking

1. Introduction.
“I am [name of experimenter], from the Psychology Department at [*omitted for blind review*]. We’re conducting a survey on morality and how people make decisions. The survey should take approximately five minutes. You will be asked to read hypothetical scenarios involving harm to people and then make several judgments. Your participation is voluntary. If you do not wish to participate, you may stop at any time. Responses will be completely anonymous; at most, you will only provide your age and gender. The survey involves thinking about hypothetical scenarios involving harm to people. If you think reading descriptions of people experiencing harm will be upsetting to you, or do not wish to read about this, you shouldn’t participate. Taking part in this survey is your agreement to participate. Do you have any questions?”

2. Perspective-taking manipulation

Close condition

Participants in the *close perspective* condition were instructed, “As you read the following, please put yourself in [Andy’s/Ellen’s/Dave’s] shoes. Imagine what life is like for [him/her] and what [she/she] feels, sees, and hears as these events take place”.

Distant condition

Participants in the *distant perspective* condition were instructed, “As you read the

following, please imagine the events from a bird's-eye perspective, as though you are looking over the scene".

3. *Suffering situation.* Within each condition, participants were randomly assigned to read one of three possible vignettes, intended to be interchangeable.

Burning building. The burning building scenario was identical to that used in Experiment 1 but with the name "Ellen" inserted as the protagonist.

Hospital emergency. The hospital emergency scenario was identical to that used in Experiment 1 but with the name Andy inserted as the protagonist and three introductory sentences added: "Andy used to be a world-class photographer for National Geographic. More than anything, he loved to travel and take pictures. However, about two years ago everything changed when he was diagnosed with life-threatening blood disease that had already damaged much of his body."

Shark attack. Participants assigned to the shark attack scenario read about a healthy man, Dave, surfing in the ocean:

> "Dave, a professional surfer, was recently signed by one of the most prominent sponsors in the country. With his lifelong dream becoming a reality, Dave intensified his practice regime by adding early morning training sessions. Surfing is Dave's sole passion; he enjoys the serenity that comes from being the only person on the water."
> "Imagine that today, while bobbing in the ocean waiting to catch a wave, Dave catches a glimpse of something heading toward him underwater. As it approaches, he realizes that it is a great white shark. Dave scrambles quickly onto the top of his board for safety, but the shark lunges at Dave, grabbing his leg and biting deeply into his flesh. Dave writhes in agony as he feels his muscles tear, the shark's sharp teeth scraping the bone. The more Dave struggles, the harder the shark thrashes against him, attacking his chest and dragging him down beneath the water's surface where he struggles for air. As Dave strains to reach for his board with one arm through the cloud of his own blood, the shark thrashes sharply, and Dave feels the skin on one side of his face become loose as he is knocked sideways. Dave's injuries are severe. If he lives, he will never surf again."

4. Life or death decision

In the *burning building* scenario, participants were asked, "All things considered, would it be better if (A) Ellen is saved by responding firefighters or (B) Ellen is killed instantly by a falling beam?

In the *hospital emergency* scenario, participants were asked, "All things considered, would it be better if (A) A technician arrives in time to repair the equipment and Andy continues living or (B) A technician does not arrive in time to repair the machine and Andy dies?"

In the *shark attack* scenario, participants were asked, “All things considered, would it be better if (A) A patrol boat sees Dave and takes him to shore alive or “(B) Dave dies before the patrol boat arrives?”

In all cases, participants made their response on a scale anchored by “A is much better” (coded as 7) and “B is much better” (coded as 1) as in Experiment 1.

5. Emotion assessment.

"How much would you say [name] is suffering *right now*?"
1;7;Very little;A moderate amount;A tremendous amount

"If [he/she] lives, how much is [name] likely to suffer *in the future*?"
1;7;Very little;A moderate amount;A tremendous amount

“How upsetting was it for you to think about [name]?”
1;7;Not at all;Moderately;Extremely

Experiment 3: Perceived Consciousness

1. Introduction (same as in Experiment 2).
“I am [name of experimenter], from the Psychology Department at [*omitted for blind review*]. We’re conducting a survey on morality and how people make decisions. The survey should take approximately five minutes. You will be asked to read hypothetical scenarios involving harm to people and then make several judgments. Your participation is voluntary. If you do not wish to participate, you may stop at any time. Responses will be completely anonymous; at most, you will only provide your age and gender. The survey involves thinking about hypothetical scenarios involving harm to people. If you think reading descriptions of people experiencing harm will be upsetting to you, or do not wish to read about this, you shouldn’t participate. Taking part in this survey is your agreement to participate. Do you have any questions?”

2. Consciousness manipulation and suffering situation.
Participants received instructions to read one of the three vignettes used in Experiment 2, but without perspective-taking instructions. Vignettes were identical across conditions until the end, at which point the consciousness of the protagonist was manipulated through a small edit to the story. The edited portion of the text appears below.

Burning building scenario:
Conscious condition: “The smoke begins to engulf her, and around her, she sees parts of the building begin to collapse.”
Unconscious condition: “The smoke engulfs her, and she passes out, unconscious. Around her, parts of the building have begun to collapse.”

Hospital emergency scenario:
Conscious condition: “…early this morning, Andy has a severe heart attack that requires immediate surgery. Andy is rushed to the operating room…”
Unconscious condition: “…early this morning, Andy has a severe heart attack and loses

consciousness. Needing immediate surgery, Andy is rushed to the operating room...”

Shark attack scenario:
Conscious condition: “the shark thrashes sharply, and Dave feels the skin on one side of his face become loose as he is knocked sideways”
Unconscious condition: “the shark thrashes sharply, and Dave feels the skin on one side of his face become loose as he is knocked unconscious”

3. Life or death decision (same as in Experiments 1 and 2).

In the *burning building* scenario, participants were asked, “All things considered, would it be better if (A) Ellen is saved by responding firefighters or (B) Ellen is killed instantly by a falling beam?

In the *hospital emergency* scenario, participants were asked, “All things considered, would it be better if (A) A technician arrives in time to repair the equipment and Andy continues living or (B) A technician does not arrive in time to repair the machine and Andy dies?”

In the *shark attack* scenario, participants were asked, “All things considered, would it be better if (A) A patrol boat sees Dave and takes him to shore alive or “(B) Dave dies before the patrol boat arrives?”

In all cases, participants made their response on a scale anchored by “A is much better” (coded as 7) and “B is much better” (coded as 1) as in Experiments 1 and 2.

4. Emotion assessment (same as in Experiment 2).

"How much would you say [name] is suffering *right now*?"
1;7;Very little;A moderate amount;A tremendous amount

"If [he/she] lives, how much is [name] likely to suffer *in the future*?"
1;7;Very little;A moderate amount;A tremendous amount

“How upsetting was it for you to think about [name]?”
1;7;Not at all;Moderately;Extremely
